# Nursing interventions to reduce mental health problems in nursing students: a scoping review

**DOI:** 10.1186/s12912-025-03329-w

**Published:** 2025-07-01

**Authors:** Iyus Yosep, Rohman Hikmat, Ai Mardhiyah, Asroful Hulam Zamroni, Yodha Pranata, Rizky Lukman Saputra

**Affiliations:** 1 Department of Mental Health, Faculty of Nursing, Universitas Padjadjaran, Sumedang, Jawa Barat Indonesia; 2 Nursing Department, Faculty of Health Science, Universitas ‘Aisyiyah Bandung, Bandung, 40264 Indonesia; 3 Department of Pediatric Nursing, Faculty of Nursing, Universitas Padjadjaran, Jawa Barat, Sumedang, Indonesia; 4 Master of Nursing Program, Faculty of Nursing, Universitas Airlangga, Surabaya, 60115 Indonesia; 5 Master of Nursing Program, Faculty of Nursing, Universitas Padjadjaran, Sumedang, West Java 45363 Indonesia

**Keywords:** Intervention, Mental health, Nursing, Students

## Abstract

**Backgrounds:**

Mental health problems among nursing students are becoming an increasingly pressing concern, given the high levels of stress and academic pressure they face. Nursing students are more susceptible to mental health challenges such as anxiety, depression, and burnout. Therefore, various interventions aimed at addressing these issues need to be further explored and mapped.

**Objective:**

This study aims to identify and map various interventions that have been implemented to address mental health problems among nursing students.

**Methods:**

This study used a scoping review design, with articles retrieved from four databases: Scopus, PubMed, Web of Science, and CINAHL. Keywords used in the search included “nursing students,” “interventions,” and “mental health.” Inclusion criteria comprised original research articles that discussed interventions without a comparison group, published in English, and within the last 10 years (2015–2025). Data were manually extracted using tables and analyzed using descriptive qualitative methods.

**Results:**

A total of 12 articles met the inclusion criteria, presenting a variety of interventions such as peer support programs, coping skills training, and mindfulness-based therapies. Findings indicated that these interventions were associated with reduced anxiety levels and improvements in the psychological well-being of nursing students. Themes that emerged from the analysis included the contribution of social support, the role of skill development, and the relevance of a holistic approach in addressing mental health concerns.

**Conclusion:**

Interventions targeting mental health concerns in nursing students have shown promising outcomes in promoting psychological well-being. Further research is recommended to explore more innovative and context-specific interventions tailored to the unique needs of nursing students.

**Supplementary Information:**

The online version contains supplementary material available at 10.1186/s12912-025-03329-w.

## Introduction

Mental health problems, such as anxiety, stress, depression, and burnout are common among nursing students. Nursing students experience higher levels of stress and anxiety than students from other study programs, due to the intensity of academic pressures that are typical in nursing education [1]. One of the main triggering factors is the heavy academic load, where students are expected to understand a large amount of theoretical material in a short time, often accompanied by rigorous exams [2]. In addition, the demands of the clinical practice they undergo add to the burden, as nursing students must integrate theory with practice in a stressful hospital environment [3]. Emotional stress is also a dominant factor, especially since nursing students are routinely confronted with situations involving patient suffering and often death [4].

Students in mental health nursing courses reported anxiety linked to course content and clinical experiences [5]. Studying nursing has very high demands which have an impact on mental health problems [2]. The nursing study program provides lectures with a tight schedule, a combination of theory and clinical assignments, written exams, and oral presentations. This program requires extra time for high learning and can be quite burdensome for students, causing fatigue and exhaustion, thus affecting mental health [6].

Nursing students feel emotionally and physically burdened because they are expected to handle medical equipment, apply theory to practice, and have direct contact with sick patients [7]. Nursing students are at high risk for emotional exhaustion, compassion fatigue, and anxiety due to continued exposure to traumatic events [8]. In addition, lack of familiarity with medical equipment, as well as limited clinical experience, can also cause fear and anxiety because students may feel anxious and afraid of making mistakes [9].

Interventions to reduce mental health problems in nursing students are essential, considering the high levels of stress, anxiety, and burnout they often experience throughout their education and clinical training [10]. Previous research has demonstrated that various approaches such as stress management training, mindfulness-based activities, peer support programs, and psychological counselling can help students develop coping skills, regulate emotions, enhance personal resilience, and manage psychological distress effectively [11, 12]. These interventions not only provide immediate relief from emotional strain but also build long-term adaptability and well-being, enabling nursing students to better navigate the academic and clinical demands of their profession [13–15].

However, despite the growing number of initiatives targeting mental health among nursing students, there remains a lack of synthesis regarding the types of interventions used, their scope, and the contexts in which they are implemented. The literature reflects a wide variation in intervention strategies ranging from individual-focused to group-based approaches, making it challenging to determine common patterns or establish comprehensive understanding [16]. Although some studies highlight positive outcomes, the diversity in methods and outcomes reported limits generalizability and comparability across settings [17].

Given these gaps, a scoping review is considered appropriate to explore and map the range of interventions aimed at reducing mental health problems in nursing students. This approach does not assess the quality or relative efficacy of the interventions, but rather provides a broad overview of what has been implemented, in what settings, and for what purposes [18, 19]. The objective of this review is to identify and map existing interventions that target mental health problems among nursing students, while highlighting key themes and identifying areas that warrant further investigation.

## Materials and methods

### Study design

This study employed a scoping review design guided by the framework developed by Arksey and O’Malley and further refined by the Joanna Briggs Institute (JBI). This design was chosen to systematically map existing evidence on interventions aimed at improving mental health in nursing students. Scoping reviews are particularly suitable for exploring broad topics and identifying gaps in the literature, especially when the body of evidence includes various study designs and outcome measures. The stages followed in this review included: (1) identifying the research question; (2) identifying relevant studies; (3) selecting studies; (4) charting the data; and (5) collating, summarizing, and reporting the results.

### Search Strategy and Eligibility Criteria

Search strategy and eligibility criteriaThe literature search was conducted systematically across four major electronic databases: Scopus, PubMed, Web of Science, and CINAHL. These databases were selected based on the Core-Standard-Ideal (CSI) model recommended by the National Library of Medicine, which emphasizes using core biomedical databases (e.g., PubMed), multidisciplinary databases (e.g., Scopus and Web of Science), and discipline-specific databases (e.g., CINAHL for nursing literature). This selection was made to ensure a balance of breadth and subject specificity (See Supplementary File 1).

The search strategy was developed using both MeSH terms and free-text keywords, and Boolean operators were applied. The search strategy was reviewed internally by two authors with expertise in literature searches; however, it was not reviewed by a professional librarian due to resource limitations. Future studies may benefit from formal consultation with an information specialist. The main keywords used included: “nursing students,” “nurse students,” “interventions,” “programs,” “strategies,” “mental health,” and “psychological well-being”. The search strategy showed in Table (Table 1):

**Table 1:** Keywords for search reports

Database	Search Strategy
Scopus	TITLE-ABS-KEY(“nursing students” OR “nurse students” OR “student nurses” OR “nursing undergraduates”) AND TITLE-ABS-KEY(“nursing care” OR “nursing interventions” OR “nursing programs” OR “nursing treatments” OR “nursing approaches”) AND TITLE-ABS-KEY(“mental health” OR “psychological health” OR “emotional well-being” OR “psychological well-being”)
PubMed	(“Nursing Students“[MeSH] OR “Nurse Students“[Tw] OR “Student Nurses”[Tw] OR “Nursing Undergraduates“[Tw] AND (“nursing care”[MeSH] OR “Nursing Interventions“[Tw] OR “Nursing programs“[Tw] OR “Nursing approaches“[Tw] OR “Nursing Treatments“[Tw]) AND (“Mental Health“[MeSH] OR “Psychological Health“[Tw] OR “Emotional Well-Being“[Tw] OR “Psychological Well-Being“[MeSH])
CINAHL	(MH “Nursing Students” OR MH “Nurse Students” OR MH “Student Nurses” OR MH “Nursing Undergraduates”) AND (MH “Nursing care” OR MH “Nursing interventions” OR MH “Nursing programs” OR MH “Nursing approaches” OR MH “Nursing Treatments”) AND (MH “Mental Health” OR MH “Psychological Health” OR MH “Emotional Well-Being” OR MH “Psychological Well-Being”)
Web of Science	TS=(“nursing students” OR “nurse students” OR “student nurses” OR “nursing undergraduates”) AND TS=(“nursing care*” OR “nursing interventions*” OR “nursing programs*” OR “nursing approaches*” OR “nursing treatments*”) AND TS=(“mental health” OR “psychological health” OR “emotional well-being” OR “psychological well-being”)

The literature search was conducted between January 1, 2025 - January 31, 2025, and all search activities were documented and reported using the PRISMA-ScR flow diagram to ensure transparency and reproducibility.

### Inclusion and exclusion criteria

In contrast to systematic reviews that typically use the PICO (Population, Intervention, Comparison, Outcome) framework, this scoping review adopted the PCC framework (Population, Concept, Context) as recommended by the JBI for scoping review methodology.

- Population: Undergraduate nursing students.
- Concept: Interventions or programs aimed at improving mental health or psychological well-being.
- Context: Higher education institutions or clinical learning environments globally.

This review included original research articles that described any type of intervention (e.g., psychosocial, educational, digital-based, or mindfulness interventions) designed to improve the mental health of nursing students. Unlike traditional scoping reviews that include all study designs, this review focused on quantitative studies with experimental elements (RCTs and quasi-experimental studies) to map interventions that were evaluated using quantitative methods (e.g., RCTs and quasi-experimental designs), while acknowledging that this review does not assess the strength or reported outcomes of the evidence. This decision was made to provide a more targeted map of rigorously evaluated interventions while acknowledging the broader scope inherent to scoping reviews.

The inclusion criteria for this review were carefully determined to ensure the relevance and quality of the selected literature. Only peer-reviewed full-text articles published between 2015 and 2025 (last 10 years) were considered, as this time frame reflects the most current practices and trends in mental health interventions for nursing students. Articles had to be written in English and contain clearly described intervention components as well as measurable outcomes related to mental health. On the other hand, several exclusion criteria were applied to refine the selection. Editorials, commentaries, study protocols, and review articles were excluded due to their lack of primary data. Studies that did not specifically focus on nursing students or were not related to mental health interventions were also excluded. Additionally, non-English publications and studies without accessible full texts were not considered for inclusion in this review.

### Data extraction

Data extraction was carried out using a structured charting form that was specifically developed by the authors to ensure consistency and comprehensiveness in capturing relevant information. The extracted data included several key elements: authors(s), year of publication, country, study design, sample characteristics (such as number of participants and demographics), type of intervention, duration and frequency of the intervention, instruments used to measure mental health outcomes, and key findings or outcomes. These elements were selected for their relevance in providing a clear understanding of the design and implementation. To maintain the rigor of the process, two reviewers independently performed the data extraction. Any discrepancies that arose were resolved through discussion, and if consensus could not be reached, a third reviewer was consulted to make the final decision.

### Quality appraisal

Although quality appraisal is not mandatory in scoping reviews, this study included an optional quality assessment using the JBI Critical Appraisal Tools to enhance transparency and guide the interpretation of findings. The tools included 13 items for RCTs and 9 items for quasi-experimental studies. Studies were not excluded based on quality scores; however, studies scoring below 70% were flagged in the results as having potential limitations. The threshold was applied not for exclusion but to support a more nuanced discussion on study quality and limitations. Two reviewers independently performed the quality assessments, and disagreements were resolved through discussion or consultation with a third reviewer. It is important to note that this quality appraisal was conducted only to guide interpretation.

### Data analysis

The data were analyzed using thematic analysis to identify patterns and recurring concepts across the reviewed interventions. The analytical process involved several stages, beginning with familiarization with the extracted data, followed by the coding of relevant concepts or characteristics related to the interventions. Subsequently, descriptive themes and subthemes were generated to capture the essence of the data. A narrative synthesis was then conducted to summarize the types of interventions and their associated outcomes in relation to the research question. The themes were identified inductively, allowing for the emergence of patterns directly from the data. To ensure the credibility and objectivity of the analysis, two authors independently verified the thematic mapping. In cases where discrepancies arose, a third authors was consulted to achieve consensus and maintain the reliability of the findings.

## Results

**Fig. 1:**
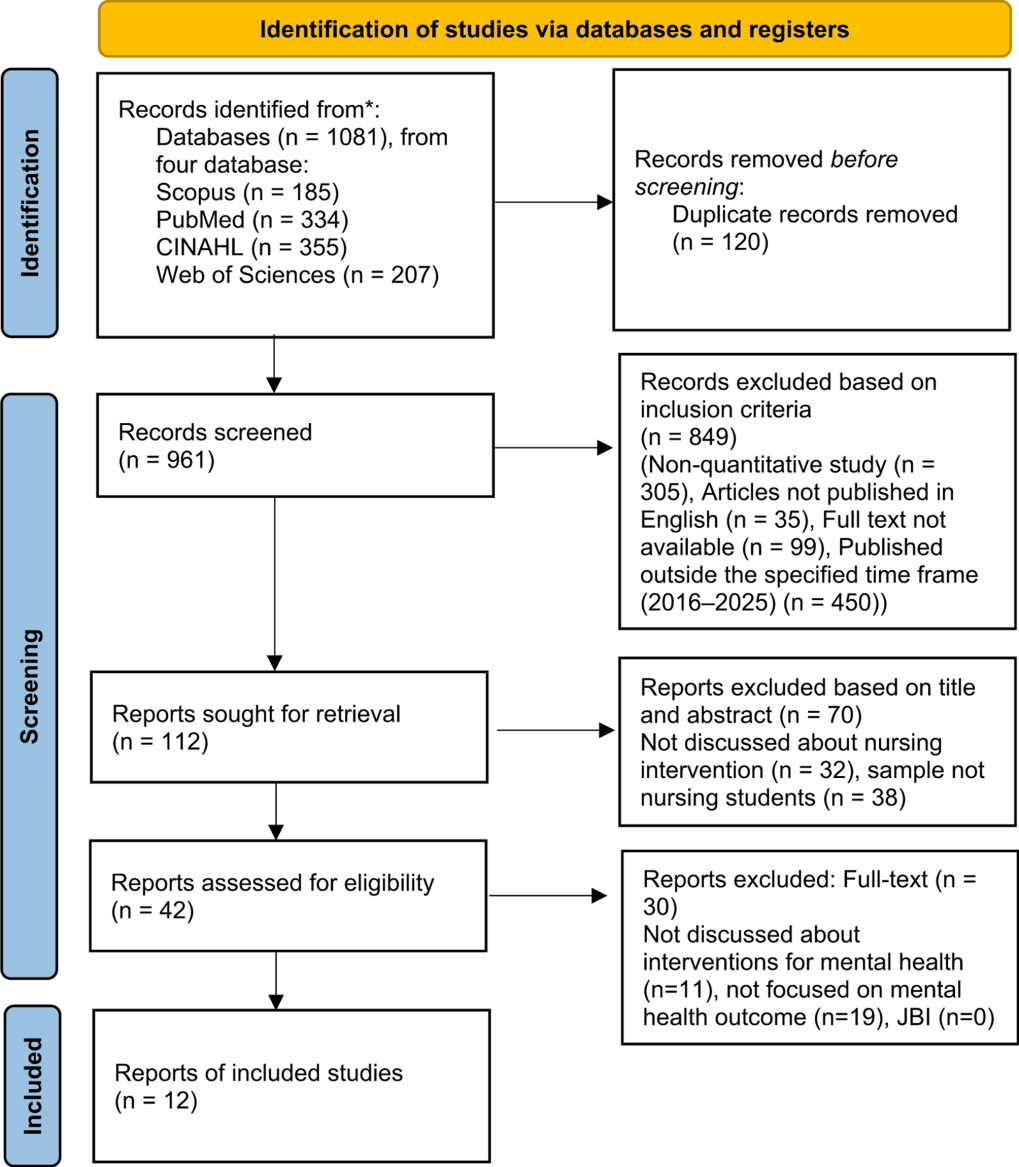
PRISMA flow diagram

An initial literature search across four electronic databases PubMed, Scopus, Web of Sciences, and CINAHL yielded a total of 1081 articles. After removing 120 duplicate records, 961 articles remained for further screening. The screening process based on titles and abstracts led to the exclusion of 849 articles that did not meet the inclusion criteria. These exclusions were due to the following reasons: 305 articles were non-quantitative studies, 35 articles were not published in English, 99 articles had inaccessible full-text versions, and 450 articles were published outside the specified time frame (2016–2025). The remaining 112 articles were sought for full-text retrieval. After assessing the full-text versions, 70 articles were excluded for the following reasons: 32 articles did not discuss nursing interventions, and 38 articles had samples that were not nursing students. The 42 articles that passed the eligibility assessment were then further reviewed. Thirty of these articles were excluded after full-text review due to the following reasons: 11 articles did not discuss interventions for mental health, and 19 articles did not focus on mental health outcomes. All 12 included articles underwent methodological quality assessment using the JBI Critical Appraisal Tools. Each study scored above 70%, indicating good methodological quality and suitability for inclusion in this review. The detailed results of the quality assessment are provided in Table 2. In the end, 12 articles met all inclusion criteria and were included in the final synthesis. A detailed summary of the article selection process is presented in Fig. 1.

**Table 2:** JBI critical appraisal tool

Author, Year	JBI critical appraisal tool	Study design
(Guo et al., 2017) [23]	92,3% (12/13)	RCT
(Ayhan & Seki Öz, 2021) [18]	88,9% (8/9)	Quasi experimental
(Song & Lindquist, 2015) [25]	92,3% (12/13)	RCT
(Vliet et al., 2017) [27]	88,9% (8/9)	Quasi experimental
(Frögéli et al., 2015) [28]	76,9% (10/13)	RCT
(İnangil et al., 2020) [19]	76,9% (10/13)	RCT
(Durgun Ozan et al., 2020) [20]	84,6% (11/13)	RCT
(Güvener, 2024) [21]	92,3% (12/13)	RCT
(Bidik & Sisman, 2024) [22]	76,9% (10/13)	RCT
(Ksiksou et al., 2023) [29]	84,6% (11/13)	RCT
(Son et al., 2019) [26]	92,3% (12/13)	RCT
(Dai et al., 2022) [24]	84,6% (11/13)	RCT

The included studies investigated a variety of interventions aimed at enhancing psychological well-being among undergraduate nursing students in different countries. The studies originated from Turkey (5 studies (41.7%)) [20–24], China (2 studies (16.7%)) [25, 26], South Korea (2 studies (16.7%)) [27, 28], the Netherlands (1 study (8.3%)) [29], Sweden (1 study (8.3%)) [30], and Morocco (1 study (8.3%)) [31]. The sample sizes across studies ranged from 50 to 208 participants. Regarding study design, ten articles (83.3%) employed randomized controlled trials (RCTs) [21–28, 30, 31], while two articles (16.7%) used quasi-experimental designs [20, 29]. The types of interventions varied in form, delivery method, and duration. Interventions included Positive Psychology Therapy (PPT) [25], Assertiveness training [20], Mindfulness-Based Stress Reduction (MBSR) [27], Mind-Body Medicine (MBM) course [29], Acceptance and Commitment Therapy (ACT) [30], music therapy and Emotional Freedom Techniques (EFT) [21], Watson’s clinical education program [22], Laughter Therapy [23], Mindful Self-Compassion (MSC) [24], Cognitive Behavioral Therapy (CBT) [31], Aromatherapy combined with Music Therapy [28], and Mindfulness Living With Challenges (MLWC) [26].

The types of interventions used in the included studies were varied, with Mindfulness-Based Interventions (such as MBSR, MSC, and MBM) being the most common, found in 5 studies (41.7%) [24–27, 29]. Cognitive-Behavioral Approaches, including ACT and CBT, were the second most frequently used, appearing in 2 studies (16.7%) [30, 31]. Laughter therapy in 1 study (8.3%) [23]. Music therapy and aromatherapy were utilized in 2 studies (16.7%) [21, 28], as well as Assertiveness training and Emotional Freedom Techniques (EFT), which were also featured in 2 studies (16.7%) [20, 21]. These interventions were delivered via face-to-face sessions, online platforms, or hybrid formats. The duration of interventions ranged from brief sessions lasting three minutes (such as EFT) to eight-week programs like MBSR and PPT. All 12 studies reported improvements across various mental health indicators, such as reduced stress, anxiety, depression, and burnout, as well as enhanced self-efficacy, resilience, empathy, self-compassion, and emotional regulation. Collectively, the studies demonstrated improvements in the psychological well-being of nursing students following these interventions. A detailed summary of the intervention outcomes is presented in Table 3.

**Table 3:** Extraction data

No	Authors, Year	Country	Purpose	Study Design	Sample	Nursing interventions	Results
1	(Guo et al., 2017) [23]	China	Assessing the effects of an 8-week PPT on nursing students’ depression and self-efficacy.	RCT	76 nursing students (mean age = 20.39)	The eight core PPT exercises include: (i) using self-strengths in a new way; (ii) three good things; (iii) gratitude visits; (iv) enjoying the moment; (v) active/constructive responses; (vi) life summaries; (vii) positive service; and (viii) maintenance. Each exercise is discussed for 1.5 h and practiced for a week. There are three seminars with 10–12 student nurses each and an instructor to facilitate the discussion.	Reduces depression and increases self-efficacy significantly (*p* < 0.05).
2	(Ayhan & Seki Öz, 2021) [18]	Turkey	Evaluation of the impact of hybrid assertiveness training on assertiveness and self-esteem.	Quasi experimental	116 students who were in their first year of undergraduate nursing education (mean age: 19.65)	Assertiveness training was conducted in person (35%) and online (65%) of the total 14 sessions. The live session lasted for 90 min, while the online session lasted for 40 min. This training covered assertiveness theory, cognitive, emotional, and behavioral aspects, and assertiveness exercises in the nursing profession.	Significantly increased assertiveness and self-esteem in the experimental group.
3	(Song & Lindquist, 2015) [25]	South Korea	Effects of MBSR on depression, anxiety, stress, and mindfulness.	RCT	44 nursing students (mean age = 19.6 years)	The MBSR program is led by instructors with over 10 years of experience and involves yoga, sitting meditation, walking, breathing techniques, body scans, and eating meditation. The program lasts for 8 weeks, 2 h each week. Participants are given homework and group discussion sessions.	Reduces depression, anxiety, stress, and increases mindfulness significantly (*p* < 0.05).
4	(Vliet et al., 2017) [27]	Netherlands	The effect of MBM course on stress, empathy, and self-reflection.	Quasi experimental	77 medical and 47 nursing students (mean age = 26.0)	The intervention in this study was a Mind-Body Medicine (MBM) conducted by six facilitators, two of whom were trained and certified in the MBM course by Georgetown University, and they trained four additional facilitators. The course consisted of five weekly sessions, each lasting four hours. Each session followed a structured format, starting with an opening ritual, such as a short meditation, followed by a check-in for sharing experiences and homework feedback. Weekly topics covered various techniques, including breathing exercises, mindfulness and loving-kindness meditation, yoga, tai-chi, guided imagery, biofeedback (autogenic training), art-based exercises, music, movement (such as dancing or exercise), and reflective writing. At the end of each session, there was a closing ritual for reflecting on experiences and a short closing meditation. Participants were given audio files of each exercise to practice at home, and they were also required to journal their experiences.	Significantly enhance empathy and imagination, also decreased personal distress.
5	(Frögéli et al., 2015) [28]	Sweden	Test of ACT intervention in reducing stress in the first semester.	RCT	113 nursing students	The six-session, two-hour program was compared to the standard reflection seminar. Students were free to choose the weekly session that fit their schedule. Students were able to attend both the reflection seminar and the intervention program even if they were not participating in the study.	Reduces stress and burnout and increases mindful awareness, effects persist on follow-up.
6	(İnangil et al., 2020) [19]	Turkey	The impact of music therapy and EFT on anxiety and vital signs.	RCT	90 nursing students (mean age = 18.9)	Music therapy was implemented in a special room with students listening to maqam mahur music for 15 min using MP3. EFT therapy was given by certified researchers by patting the participants’ body parts. Each EFT therapy session lasted three minutes.	Significantly reduced anxiety in the experimental group.
7	(Durgun Ozan et al., 2020) [20]	Turkey	The influence of Watson’s clinical education program on coping with anxiety.	RCT	103 nursing students	The intervention and control groups were placed in different clinics to avoid influencing communication between students and instructors. Instructors in the intervention group followed guidelines based on Watson’s Human Caring Theory.	Significant differences in eating patterns, sleep, and social media influence; without significant differences in learning interest.
8	(Güvener, 2024) [21]	Turkey	The effects of laughter therapy on self-efficacy and psychological well-being.	RCT	208 Nursing Students	Each laughter therapy session includes clapping, warm-ups, breathing exercises, games, and laughter exercises.	Significantly improved self-efficacy and psychological well-being.
9	(Bidik & Sisman, 2024) [22]	Turkey	The impact of the Mindful Self-Compassion program on mental health.	RCT	80 Nursing Students	The intervention was a Mindful Self-Compassion program integrated with the caritas processes of the Human Caring Theory. It consisted of six 90-minute online sessions via Zoom, focusing on mindfulness and self-compassion practices. Each session covered different topics, such as loving-kindness, self-compassion, mindful breathing, and compassion in daily life. Participants were encouraged to create a healing environment, and after each session, they received meditation and audio recordings for home practice. The program was delivered over six weeks, and pre- and post-tests were administered to assess its impact.	Significantly reduced health-promoting and protective behaviors, self-care perception, psychological resilience, and self-compassion
10	(Ksiksou et al., 2023) [29]	Morocco	The impact of CBT group therapy on internet addiction and mental health.	RCT	60 Nursing Students	The intervention group participated in several sessions of cognitive-behavioral group therapy (CBGT) that included cognitive restructuring, virtual exposure, mindfulness meditation, relationship skills development, activity planning, and relaxation techniques.	Significantly reduced depression, internet addiction, anxiety, and stress (*p* < 0.05).
11	(Son et al., 2019) [26]	South Korea	Assessing the effects of aromatherapy combined with music therapy on nursing students’ anxiety, stress, and performance of fundamental nursing skills.	RCT	98 Nursing Students	The intervention involved three groups: aromatherapy, music therapy, and a combination of both. In the aromatherapy group, participants inhaled a 1:1 mixture of Origanum majorana and Citrus sinensis essential oils for 20 min before the test. The music therapy group listened to Beethoven’s Moonlight Sonata for 20 min before the test. The combined group experienced both aromatherapy and music therapy for the same duration.	The combined aromatherapy and music therapy group showed a significant reduction in test anxiety, state anxiety, and stress, and also improved performance in fundamental nursing skills.
12	(Dai et al., 2022) [24]	China	Evaluation of the impact of the “Mindfulness Living With Challenges (MLWC)” online course on mental health, focusing on depression, anxiety, stress, and mindfulness among undergraduate nursing students during the COVID-19 pandemic.	Randomized Controlled Trial	120 Nursing Students	Participants took part in a six-week online course called “Mindfulness Living With Challenges (MLWC)”, which consisted of six sessions with two lessons per session. The course involved mindfulness meditation, mindful stretching, and walking practices, among others.	Significant reductions in anxiety and stress, along with improvements in mindfulness and perceived social support

### Psychological intervention

The study has shown that various types of psychological interventions can lead to improvements in reducing levels of depression, anxiety, and stress among nursing students. For example, a Positive Psychotherapy (PPT) program in China resulted in a significant decrease in depressive symptoms and an increase in self-efficacy (*P* < 0.05) [25]. Similarly, the Mindfulness-Based Stress Reduction (MBSR) intervention resulted in reductions in depression, anxiety, and stress while increasing mindfulness awareness (*P* < 0.05). The Acceptance and Commitment Training (ACT) intervention also led to reductions in stress and an increase in mindfulness awareness among nursing students [30].

### Diverse training methods

The assertiveness training program for nursing students included 14 sessions delivered both in person (35%) and online (65%), with in-person sessions lasting 90 min and online sessions 40 min [20]. This training covered assertiveness theory along with cognitive, emotional, and behavioral components tailored to nursing contexts. Students learned how assertive behavior supports professional communication, with practical exercises to build confidence and apply assertiveness in nursing-specific situations. By the end of the program, participants were reported to have developed improved communication skills, enhanced their ability to advocate for patients, and fostered better interpersonal interactions within clinical environments.

Mindfulness-Based Psychological Interventions involves mindfulness techniques such as meditation, deep breathing, and mindfulness [27]. Students are trained to focus on the present moment experience without judgment, which can help reduce stress and anxiety. Sessions can be conducted individually or in groups, and typically include breathing exercises, guided meditations, and reflective discussions about participants’ experiences.

The revised course incorporates kindness meditation in place of forgiveness meditation, aiming to enhance compassion and emotional resilience. Additionally, yoga or tai-chi exercises are included to support physical and mental relaxation, improve flexibility, and foster a sense of grounding and calm [29]. Students receive audio recordings for guided practice at home, enabling them to reinforce skills learned in sessions. They are also asked to maintain a journal, documenting their experiences and reflections on each practice, which helps track progress and deepen personal insights. This program is facilitated by a team of six instructors with expertise in meditation and physical exercises. The course is structured into five weekly sessions, each lasting four hours, offering a blend of meditation, physical practice, and reflective exercises designed to holistically support students’ emotional and physical well-being.

Music therapy was conducted in a designated room where students engaged in a 15-minute session of listening to maqam mahur music, facilitated through MP3 playback. This specific genre of music was chosen for its soothing and uplifting qualities, aiming to create a calming environment conducive to relaxation and emotional expression [21]. In addition to music therapy, participants also received Emotional Freedom Techniques (EFT) therapy, administered by certified authors. During each EFT session, which lasted three minutes, participants engaged in a tapping protocol where they gently tapped on specific points of their body. This method is designed to help release emotional distress and promote a sense of well-being. Together, these therapeutic approaches aimed to enhance the overall mental health and emotional resilience of the students [28]. Each laughter therapy session is designed to foster a joyful and relaxed atmosphere, incorporating a variety of engaging activities. The session begins with warm-up exercises that encourage participants to loosen up and prepare for the experience [23]. This is followed by clapping and breathing exercises to enhance relaxation and focus. Participants then engage in interactive games that promote laughter and social connection, creating a playful environment. The session culminates in laughter exercises, where individuals are encouraged to laugh freely, whether through guided laughter or spontaneous expression. These elements work together to alleviate stress, boost mood, and foster a sense of community among participants, ultimately enhancing their emotional well-being.

Participants engaged in the six-week online course titled “Mindfulness Living With Challenges (MLWC),” which comprised six sessions, each featuring two lessons. This structured program was designed to help individuals cultivate mindfulness practices that can be applied in their daily lives, particularly in navigating various challenges. Each session provided participants with valuable insights and practical exercises aimed at enhancing their mindfulness skills, promoting emotional resilience, and fostering a deeper understanding of their thoughts and feelings [26]. The online format allowed for flexibility and accessibility, enabling participants to learn and practice mindfulness at their own pace while benefiting from a supportive virtual learning environment.

### Influence of learning environment

The learning environment also plays an important role in students’ mental health. Research using Watson’s caring theory shows that clinical education programs based on the theory can reduce anxiety levels and improve students’ coping skills [22]. This suggests that teaching that focuses on empathy and caring can affect the psychological well-being of nursing students [24].

### Mental health awareness

A Moroccan study on Cognitive-Behavioral Group Therapy (CBGT) showed that awareness of addictive behavior towards the internet can be addressed through structured group therapy [31]. This finding suggests the need for programs that increase awareness of mental health and adaptive behavior among nursing students, especially in today’s digital era.

### Research gap

Based on the thematic analysis, several significant research gaps in psychological interventions to improve mental health in nursing students have been identified. First, many studies do not consider demographic variables, such as gender and age, which could potentially influence the outcomes of interventions. Additionally, most studies tend to focus on specific types of interventions, such as mindfulness training, without exploring more holistic approaches that include physical or social elements. The research methodologies used also vary, with many studies involving small sample sizes and lacking rigorous designs, which may limit the generalizability of their findings. Furthermore, the integration of psychological interventions into nursing education curricula remains limited, highlighting the need to explore the barriers that institutions encounter when attempting to implement these interventions.

## Discussion

In this scoping review, the authors aim to explore various nursing interventions designed to reduce mental health problems among nursing students. Mental health is a crucial aspect that can affect the quality of life and academic performance of students, especially in the nursing field which often faces high pressure [32]. Nursing students are not only required to have clinical knowledge and skills, but must also be able to manage stress and emotions in facing the challenges of education and clinical practice [33]. High levels of anxiety, depression, and stress among nursing students can negatively impact learning and social interactions, and potentially reduce the quality of health care in the future [34].

The findings show that psychological interventions are significantly in reducing symptoms of depression, anxiety, and stress among nursing students. For example, intervention programs such as Positive Psychotherapy (PPT) and Mindfulness-Based Stress Reduction (MBSR) have been shown to reduce levels of depression and increase awareness of mindfulness. Previous studies have shown a significant decrease in anxiety symptoms among students after participating in similar interventions [35]. These studies indicate that a structured psychological approach can help students cope with academic and emotional stress. Good mental health not only impacts the individual, but also affects overall academic performance. Other studies have shown that nursing students with high mental health problems tend to have lower academic achievement, difficulty concentrating, and higher absenteeism rates [36]. This suggests that poor mental health can create a negative cycle that impacts the learning process and development of professional skills.

The variety of intervention methods studied showed a variety of approaches in addressing mental health problems among nursing students. Some prominent interventions include Positive Psychotherapy (PPT) and Mindfulness-Based Stress Reduction (MBSR). PPT focuses on developing individual strengths and positive experiences, which can provide students with tools to build mental resilience [37]. The advantage of PPT lies in its ability to increase self-efficacy and provide a positive view of the future [38]. However, this approach may be less effective for individuals who are more focused on solving concrete problems, as PPT is more introspective. On the other hand, MBSR teaches relaxation and mindfulness techniques, which have been shown to be effective in reducing anxiety and improving overall well-being [39]. The advantage of MBSR lies in its flexibility; it can be applied in a variety of contexts, both individually and in groups. However, some studies have shown that MBSR requires a significant time commitment and ongoing practice to achieve optimal results, which can be a challenge for students with busy academic schedules [40].

Cognitive Behavioral Therapy (CBT) is a psychotherapy approach that focuses on the relationship between thoughts, emotions, and behavior. CBT helps individuals identify and change negative thought patterns that may contribute to mental health problems, such as depression and anxiety. Previous research has shown that CBT interventions can significantly reduce symptoms of depression and anxiety in a variety of populations, including patients with severe mental health problems [41]. Previous study have found that CBT has higher reported outcomes compared to other therapies in treating anxiety, with remission rates reaching 50% [36]. CBT can be implemented through individual or group counseling sessions, where individuals are taught techniques such as goal setting, problem solving, and emotional regulation exercises. Previous research has highlighted the success of implementing CBT in emotional support programs in academic settings, showing increased coping skills and decreased stress levels in participants [42, 43].

Social support plays a vital role in mental health interventions, where interactions between individuals can provide a sense of belonging and reduce feelings of isolation. Research shows that group support is effective in enhancing psychological well-being and improving overall mental health [44]. Previous studies have found that individuals who engage in group support sessions experience significant reductions in depressive and anxiety symptoms compared to those who do not receive social support [45]. Group support sessions allow individuals to share experiences and challenges they face in a safe and supportive environment. Interaction within a group can increase self-confidence and social skills, which contribute to improved mental health [46]. Through mutual support, individuals not only feel more connected, but also gain new perspectives that can help them cope with the problems they face [47].

The use of digital technologies in mental health interventions has become increasingly important, especially in the context of college students who often face significant academic and emotional challenges. Mental health apps and telehealth platforms offer accessible and flexible solutions to support psychological well-being. Previous research has shown that smartphone-based apps can help users monitor their mood and implement stress management techniques in real-time, providing interventions that are more responsive to individual needs [48, 49]. The main advantage of this technology-based method is its accessibility; students can access these resources anytime and anywhere, which is very beneficial for those with busy schedules. While several studies have shown the reported outcomes of apps in improving mental health, challenges remain such as lack of interaction, lack of support, and lack of monitoring during the intervention [44, 50]. The lack of direct interaction with a mental health professional in a telehealth context may reduce the reported outcomes of the intervention, especially for individuals who prefer face-to-face support [38].

A supportive learning environment has a significant impact on students’ mental health, especially in the context of higher education. Previous studies have shown that a positive learning atmosphere can improve psychological well-being and minimize stress levels, thereby contributing to better academic outcomes. An environment that promotes collaboration, open communication, and emotional support can provide a sense of security and increase learning motivation [51, 52]. The application of caring theory in nursing education emphasizes the importance of deep interpersonal relationships between educators and students [44, 53]. Teaching that focuses on empathy and attention to students’ emotional needs can create more inclusive and supportive learning spaces [54, 55]. Educational programs that integrate caring aspects, such as reflection sessions, peer support, and interpersonal skills training, can help students feel valued and cared for, which in turn can reduce anxiety and increase their engagement in the learning process [56, 57].

Mental health awareness is essential among students, especially in the context of nursing education, where they are expected to not only care for patients physically but also understand and support the psychological aspects of health. Research shows that mental health education can reduce the stigma often attached to mental health problems and encourage individuals to seek help when needed [47]. Another study found that increased awareness of mental health was associated with reduced addictive behaviors, such as drug use and alcohol abuse [54]. These findings indicate that by providing comprehensive education on the risk factors and impacts of mental health, students are more likely to develop adaptive coping strategies and avoid risky behaviors. In addition, a better understanding of mental health can strengthen their empathy and sensitivity skills when interacting with patients, making them more effective in providing holistic care [58].

The methodological process of this review adhered to the Arksey and O’Malley framework, encompassing the stages of identifying the research question, systematically searching for relevant literature, selecting studies based on predetermined inclusion and exclusion criteria, charting key data, and synthesizing the findings. A rigorous and transparent search strategy was implemented across multiple databases to enhance reproducibility and reduce selection bias. Although the number of studies included was relatively limited, careful data extraction and management ensured consistency and credibility of the findings. Future reviews are encouraged to incorporate formal quality appraisal of included studies and, where feasible, undertake comparative or sensitivity analyses to further strengthen the robustness of the evidence base.

Despite the limited scope of available studies, the findings of this review provide valuable insights with practical and educational implications. Psychological interventions such as Positive Psychotherapy, Mindfulness-Based Stress Reduction, and Cognitive Behavioral Therapy offer distinct mechanisms to address mental health concerns among nursing students. Furthermore, the integration of social support systems, digital-based interventions, supportive learning environments, and mental health literacy initiatives were identified as crucial components of a comprehensive approach. To maximize impact, nursing education curricula should consider incorporating multimodal interventions tailored to the diverse needs of students. Embedding mechanisms for continuous evaluation and feedback into these programs would further ensure their adaptability and long-term reported outcomes.

Future research should address several notable gaps identified in this review. There is a critical need for studies that examine the moderating effects of demographic variables such as gender, socioeconomic status, cultural background, and academic stage on intervention outcomes. Longitudinal research designs are warranted to evaluate the sustainability of intervention effects over time and their influence on students’ transition into professional practice. Additionally, comparative studies exploring the relative reported outcomes of face-to-face versus digital modalities could yield important insights into optimizing mental health interventions for contemporary nursing students. Expanding the scope of outcomes to include professional competencies, clinical performance, and patient care quality may also provide a more comprehensive understanding of the long-term benefits of mental health interventions within nursing education.

## Conclusion

This scoping review analyzed eleven articles examining interventions aimed at improving mental health among college students across various countries. The analysis identified six central themes: the reported outcomes of psychological interventions, diversity in intervention methods, the prominence of cognitive behavioral therapy, the role of group-based support, the integration of digital technologies, and the influence of academic environments. The findings demonstrated that interventions such as Positive Psychotherapy and Mindfulness-Based Stress Reduction were effective in alleviating symptoms of depression, anxiety, and stress, while also enhancing overall psychological well-being. Furthermore, the importance of social support systems and the potential of digital-based interventions were underscored as critical components in promoting student mental health.

From a nursing perspective, these findings highlight the need for nurses and nursing educators to take an active role in addressing the mental health needs of students. This could involve the development of training initiatives that incorporate evidence-based psychological strategies into nursing education, alongside the provision of sustained emotional and social support frameworks within academic settings.

Future research should prioritize investigating the moderating effects of demographic variables such as gender, cultural background, and socioeconomic status on the outcomes of mental health interventions. Additionally, there is a need for studies exploring comprehensive integration of mental health support into the nursing curriculum, thereby equipping future nurses with both the skills to manage their own psychological well-being and to support the mental health of their patients.

## Electronic supplementary material

Below is the link to the electronic supplementary material.

Supplementary Material 1

## Data Availability

All data generated or analysed during this study are included in this published article.
