# Supplementary material for: Nursing interventions to reduce mental health problems in nursing students: a scoping review
Source: BMC Nurs. 2025 Jul 1;24:780. doi: 10.1186/s12912-025-03329-w (PMC12211802; doi:10.1186/s12912-025-03329-w)
Supplement: Supplementary file 1 — Supplementary Material 1 [file 12912_2025_3329_MOESM1_ESM.docx]

**Supplementary File 1. Searching Strategy**

**Title**: Nursing Interventions to Reduce Mental Health Problems in Nursing Students: A Scoping Review

**Concept 1: Nursing Students**

- **Keywords**: nursing students, nurse students, student nurses, nursing undergraduates
- **MeSH (PubMed)**: "Nursing Students"[MeSH]
- **Combined**: "Nursing Students"[MeSH] OR "nursing students"[tw] OR "nurse students"[tw] OR "student nurses"[tw] OR "nursing undergraduates"[tw]

**Concept 2: Nursing Interventions**

- **Keywords**: nursing care, nursing interventions, nursing programs, nursing treatments, nursing approaches
- **MeSH (PubMed)**: "Nursing Care"[MeSH]
- **Combined**: "Nursing Care"[MeSH] OR "nursing care"[tw] OR "nursing interventions"[tw] OR "nursing programs"[tw] OR "nursing treatments"[tw] OR "nursing approaches"[tw]

**Concept 3: Mental Health**

- **Keywords**: mental health, psychological health, emotional well-being, psychological well-being
- **MeSH (PubMed)**: "Mental Health"[MeSH], "Psychological Well-Being"[MeSH]
- **Combined**: "Mental Health"[MeSH] OR "Psychological Health"[tw] OR "Emotional Well-Being"[tw] OR "Psychological Well-Being"[MeSH]

**Table of Search Terms PubMed**

| **Concept 1** | **Concept 2** | **Concept 3** |
| --- | --- | --- |
| "Nursing Students"[MeSH] OR "Nurse Students"[Tw] OR "Student Nurses”[Tw] OR "Nursing Undergraduates"[Tw] | "nursing care”[MeSH] OR “Nursing Interventions"[Tw] OR "Nursing programs"[Tw] OR "Nursing approaches"[Tw] OR "Nursing Treatments"[Tw] | "Mental Health"[MeSH] OR "Psychological Health"[Tw] OR "Emotional Well-Being"[Tw] OR "Psychological Well-Being"[MeSH] |

**Table of Search Terms SCOPUS**

| **Concept 1** | **Concept 2** | **Concept 3** |
| --- | --- | --- |
| TITLE-ABS-KEY("nursing students" OR "nurse students" OR "student nurses" OR "nursing undergraduates") | TITLE-ABS-KEY("nursing care" OR "nursing interventions" OR "nursing programs" OR "nursing treatments" OR "nursing approaches") | TITLE-ABS-KEY("mental health" OR "psychological health" OR "emotional well-being" OR "psychological well-being") |

**Table of Search Terms WoS**

| **Concept 1** | **Concept 2** | **Concept 3** |
| --- | --- | --- |
| TS=("nursing students" OR "nurse students" OR "student nurses" OR "nursing undergraduates") | TS=("nursing care*" OR “nursing interventions*" OR "nursing programs*" OR "nursing approaches*" OR "nursing treatments*") | TS=("mental health" OR "psychological health" OR "emotional well-being" OR "psychological well-being") |

**Table of Search Terms CinaHl**

| **Concept 1** | **Concept 2** | **Concept 3** |
| --- | --- | --- |
| MH "Nursing Students" OR MH "Nurse Students" OR MH "Student Nurses" OR MH "Nursing Undergraduates" | MH “Nursing care” OR MH "Nursing interventions" OR MH "Nursing programs" OR MH "Nursing approaches" OR MH "Nursing Treatments" | MH "Mental Health" OR MH "Psychological Health" OR MH "Emotional Well-Being" OR MH "Psychological Well-Being" |

**Table of Article Screened**

| **Screening Stage** | **SCOPUS** | **PUBMED** | **WOS** | **CINAHL** | **TOTAL** |
| --- | --- | --- | --- | --- | --- |
| **Records identified from databases** | 185 | 334 | 207 | 355 | 1081 |
| **Records removed before screening**  └ Duplicate records removed | – | – | – | – | (n = 120) |
| **Records screened** | – | – | – | – | **961** |
| └ Records excluded based on inclusion criteria (n = 849) | | | | | |
| • Non-quantitative studies | – | – | – | – | (n = 305) |
| • Not published in English | – | – | – | – | (n = 35) |
| • Full text not available | – | – | – | – | (n = 99) |
| • Outside time frame (2016–2025) | – | – | – | – | (n = 450) |
| **Reports sought for retrieval** | – | – | – | – | **112** |
| └ Reports excluded (title/abstract review) (n = 70) | | | | | |
| • Not about nursing interventions | – | – | – | – | (n = 32) |
| • Sample not nursing students | – | – | – | – | (n = 38) |
| **Reports assessed for eligibility** | – | – | – | – | **42** |
| └ Reports excluded (full-text review) (n = 30) | | | | | |
| • Not discussing MH interventions | – | – | – | – | (n = 11) |
| • Not focused on MH outcomes | – | – | – | – | (n = 19) |
| **Reports of included studies (synthesized)** | – | – | – | – | **12** |
